# Supplementary material for: Off-the-shelf proximity biotinylation for interaction proteomics
Source: Nat Commun. 2021 Aug 18;12:5015. doi: 10.1038/s41467-021-25338-4 (PMC8373943; doi:10.1038/s41467-021-25338-4)
Supplement: Supplementary file 1 — Supplementary Information [file 41467_2021_25338_MOESM1_ESM.pdf]

Supplementary Information

'Off the shelf' proximity biotinylation for interaction proteomics

Irene Santos-Barriopedro[1] #, Guido van Mierlo[1] #* and Michiel Vermeulen[1] *

[1]Department of Molecular Biology, Faculty of Science, Radboud Institute for Molecular Life Sciences, Oncode Institute, Radboud University Nijmegen, the Netherlands.

# These authors contributed equally

* Correspondence to: guido.vanmierlo@epfl.ch and michiel.vermeulen@science.ru.nl

Supplementary Figure 1

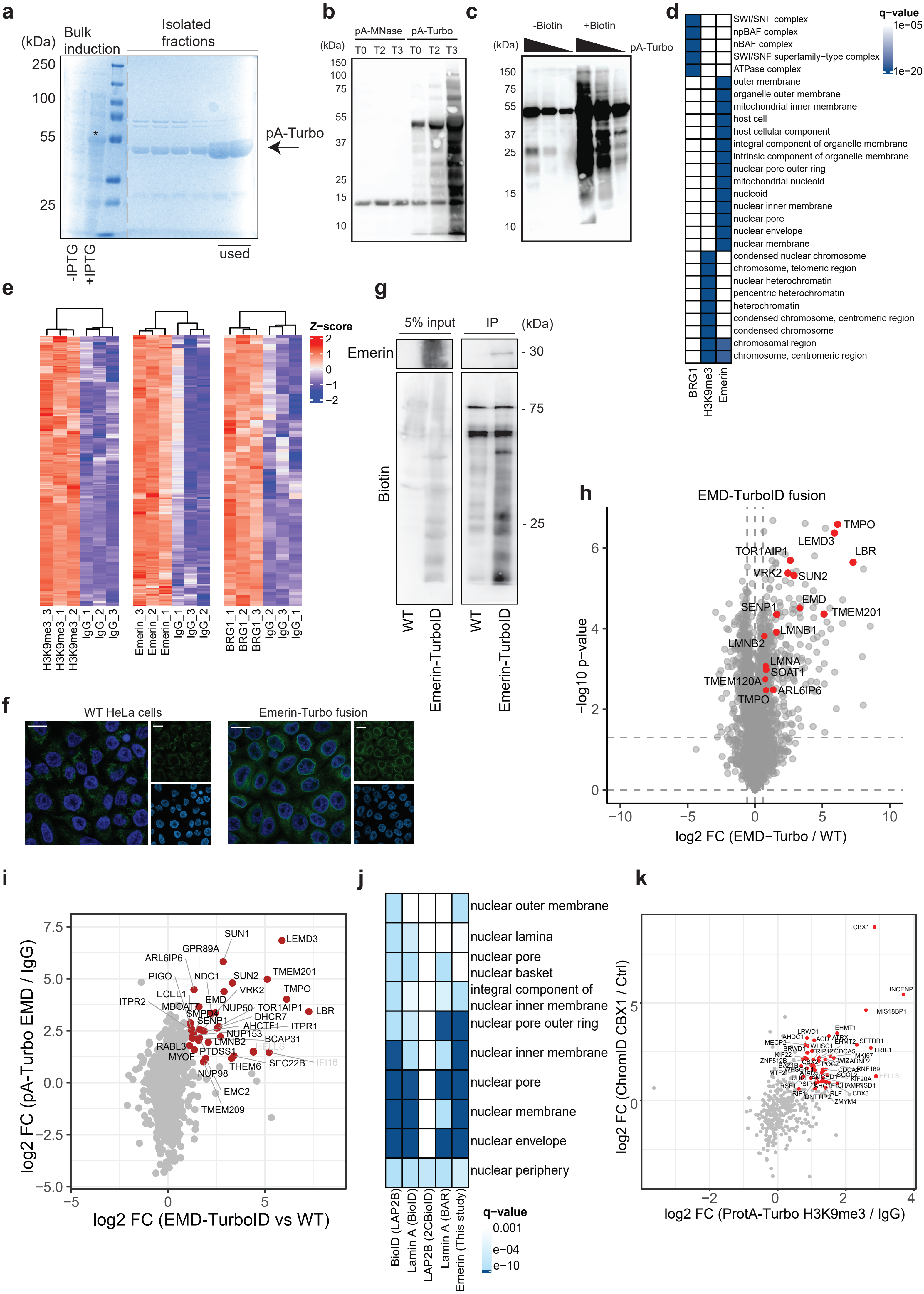

Supplementary Figure 1: Validation of ProtA-Turbo targeting of sub-nuclear compartments in fixed HeLa cells. (**a**) Coomassie staining of the recombinantly produced ProtA-TurboID fusion protein. Bulk induction indicates bacteria lysates in which IPTG induced protein of the ProtA-Turbo enzyme (asterisk). 'Used' indicates the fractions with the ProtA-Turbo that was used in this study. (**b**) ProtA-Turbo retains biotinylation activity in bacteria (T is hours after induction, all conditions have biotin in the medium). ProtA-MNase was used as a control. (**c**) ProtA-Turbo displays strong biotinylation activity when added to cell extracts. (**d**) GO-terms associated with proteins enriched in ProtA-Turbo targeting of BRG1, Emerin and H3K9me3. q-values represent p-values adjusted for multiple testing. (**e**) Heatmap showing the enrichment of proteins in each of the three replicates visualized in the volcano plots in Figure **1d**. (**f**) Biotinylation signal in wildtype HeLa or HeLa cells containing a genetically-engineered Emerin-TurboID fusion protein. Scale bars represent 10 µm. (**g**) Biotin-IP of the cells in (f). (**h**) Volcano plot of mass spectrometry analyses of the cells in (f) (n=3). Highlighted proteins represent a selection of known lamin-associated proteins. (**i**) Scatter plot of the fold-change over control of TurboID-Emerin fusion and Emerin targeted with ProtA-Turbo. (**j**) GO terms associated with proteins enriched using different lamina targeting strategies. q-values represent p-values adjusted for multiple testing. (**k**) Scatter plot of the fold-change over control of ChromID using a CBX1 reader domain (thus targeting (pericentric) heterochromatin) versus H3K9me3 targeting using ProtA-Turbo. Number of independent experiments performed: **a** 2; **b** 2; **c** 4; **f** 2; **g** 2; **h** 2, n=3 replicates per independent experiment. Source data are provided as a Source Data file.

Supplementary Figure 2

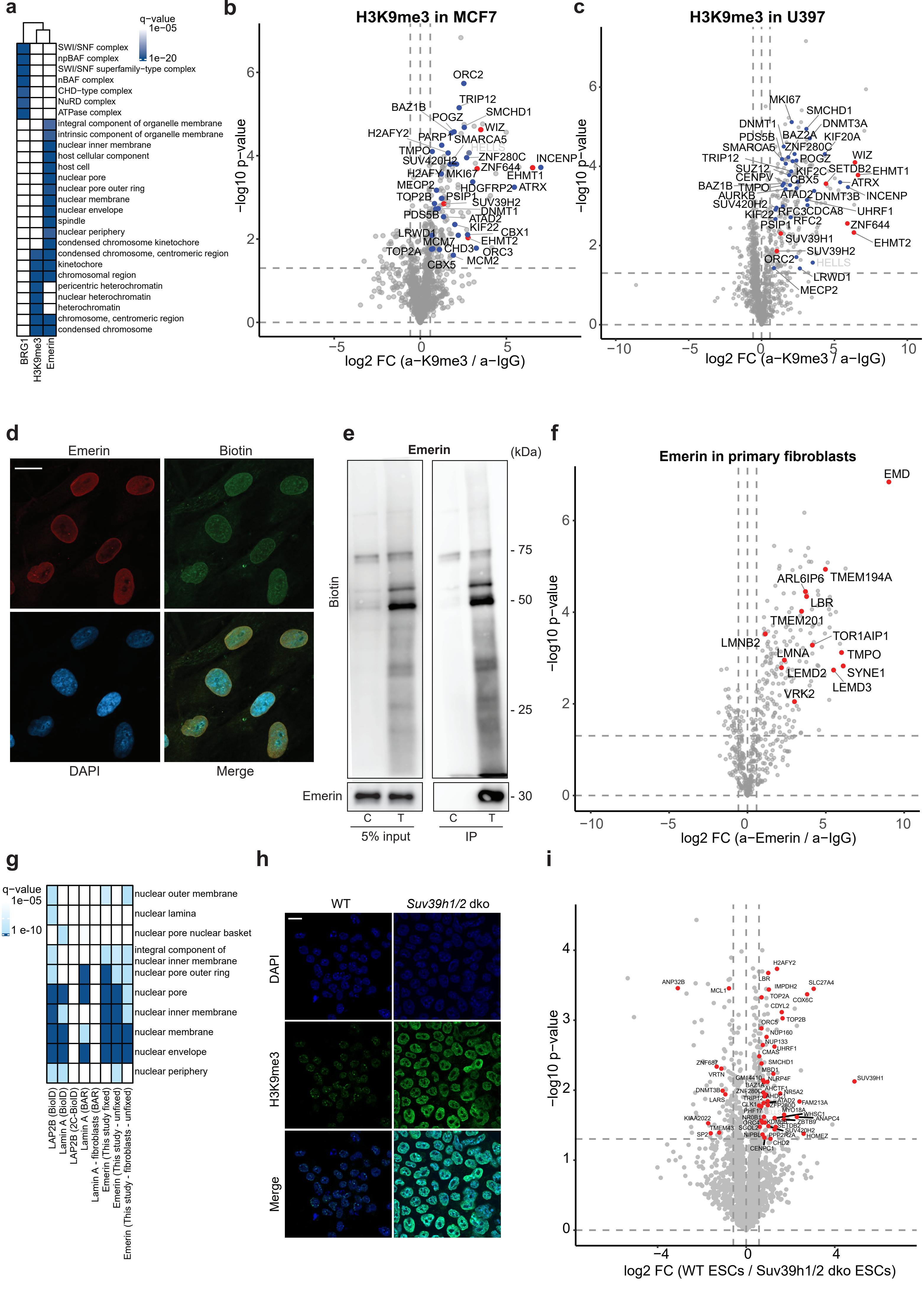

Supplementary Figure 2: Validation of ProtA-Turbo targeting of sub-nuclear compartments in several cell types. (**a**) GO-terms associated with proteins enriched in ProtA-Turbo targeting of BRG1, Emerin and H3K9me3. q-values represent p-values adjusted for multiple testing. (**b**) Volcano plots of mass spectrometry analyses of biotin IPs after targeting H3K9me3 or IgG in fixed MCF7 cells (n=3). Red: H3K9me3 writers / writer complexes. Blue: selection of known (peri)centric heterochromatin binding proteins. Protein names in white indicate potential streptavidin contaminants (see also Source Data). (**c**) Volcano plot of proteomics analyses after H3K9me3 targeting using ProtA-Turbo in unfixed U937 cells (n=3). Red: H3K9me3 writers / writer complexes. Blue: selection of known (peri)centric heterochromatin binding proteins. Protein names in white indicate potential streptavidin contaminants (see also Source Data). (**d**) Immunofluorescence of Emerin targeting in primary, low-passage, human fibroblasts. Scale bars indicate 10 µm. (**e**) Biotin IPs of the same cells as in (d). (**f**) Proteomics analyses of the same cells as in (d). A selection of known lamina-associated proteins is highlighted. (**g**) GO-terms as in (b), now including targeting of primary human fibroblasts in this study and in a previous study using BAR. q-values represent p-values adjusted for multiple testing. (**h**) Immunofluorescence images of H3K9me3 (green) and DAPI staining (blue) in wildtype and *Suv39h1/2* dko ESCs. Scale bars indicate 10 µm. (**i**) ProtA-Turbo targeting of H3K9me3 followed by mass spectrometry of the same cells as in (h). Number of independent experiments performed: **b** 2, n=3 replicates per independent experiment; **c** 1, n=3 replicates; **d** 2; **e** 2; **f** 2, n=3 replicates per independent experiment; **h** 2; **i** 2, n=3 replicates per independent experiment. Source data are provided as a Source Data file.

Supplementary Figure 3

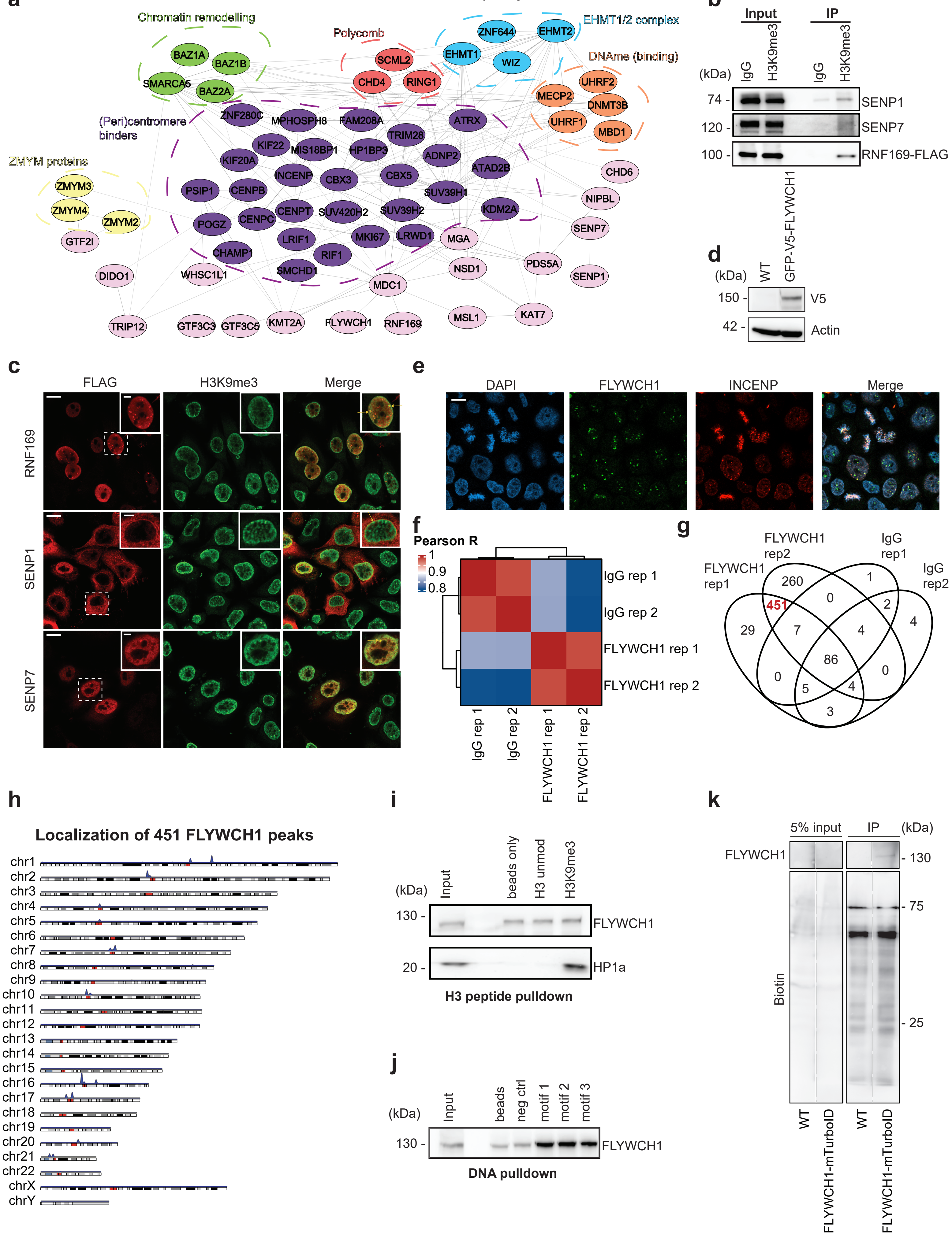

Supplementary Figure 3: Identification of FLYWCH1 as a (peri)centric heterochromatin binder. (**a**) Interaction network of proteins identified in at least 3 out of 4 H3K9me3 targeting experiments (HeLa cells fixed, HeLa cells unfixed, MCF7 cells fixed and U937 unfixed). All proteins highlighted are either experimentally validated or in a database found as interacting with at least one other protein in the network (interaction links are visualized as grey lines). Light-pink nodes represent candidate H3K9me3-marked heterochromatin binders. (**b**) protA-Turbo IPs followed by western blot validation of three candidate H3K9me3-proximal proteins. (**c**) Immunofluorescence of the same proteins as in (b) expressed with a FLAG tag (red) and co-stained for H3K9me3 (green). Yellow arrows in the zoomed in panels of one cell indicate regions of overlap between the candidate proteins and H3K9me3. Scale bars indicate 10 µm (large panels) or 3 µm (small panels). (**d**) Western blot validation of endogenous tagging of FLYWCH1 with a GFP-V5 tag. (**e**) IF staining for INCENP and FLYWCH1 in synchronized HeLa cells. Scale bars indicate 10 µm. (**f**) Correlation of the signal under peaks for FLYWCH1 and IgG ChIPs. (**g**) Number of called peaks for FLYWCH1 and IgG. The 451 peaks highlighted in red were used for further analyses. (**h**) Genome-wide coverage plot showing the localization of the 451 FLYWCH1-specific peaks on the human genome. (**i**) Peptide pulldown using either the unmodified H3 tail or the same peptide containing the H3K9me3 modification. Beads only without a peptide immobilized to it were used as a control. Input represents 5% of the input extract. (**j**) DNA pulldown followed by western blot for FLYWCH1-V5-GFP using an anti-V5 antibody. Input represents 5% of the input extract. Beads represent the bead only control, negative ctrl is a scrambled version of the DNA motifs in Figure 3b and motif1-3 represent different versions of the motifs in Figure 3b. (**k**) Western blot validation of the endogenous FLYWCH1-miniTurboID fusion protein. The dashed line indicates that for visual purposes one western blot lane in between has been removed. Number of independent experiments performed: **b** 2; **c** 2; **d** 2; **e** 2; **i** 2; **j** 2; **k** 1.

Supplementary Table 1. Primers and gBlocks used in this study

**Cloning primers and gBlocks**	
TurboEcoRI-F	CCGAATTCGCTAGCAAAGACAATACTGTG
TurboBamHIfrag-R	GGCCAGCTCCACCAGGATCCCTGCCAGCT
TurboBamHIfrag-F	AGCTGGCAGGGATCCTGGTGGAGCTGGCC
TurboBamHI-R	CCGGATCCTCAGTCGGCCCTGCTGAATTCCTTTTCG
6xHistagHinDIII-F	AGCTTCATGCATCATCACCACCACCAT
6xHistagHinDIII-R	AGCTATGGTGGTGGTGATGATGCATGA
HA1emerinMluI-Fw	CCGACGCGTTAGTGGCGTCCGGGCTCGCAGTAC
HA1emerinNcoI-Rv	GGCCATGGGGCCTACCAAAACCGGCCGAGCAACG
HA2emerinHindIII-Fw	CCAAGCTTGCATGGACAACTACGCAGATCTTTCGGA
HA2emerinAscI-Rv	GAAGGCGCGCCCCAACCCCTGCCTTACCCTTGCTCTG
gRNAEmerinFw2	CACCGCGCCCACGCCCGAGTCCGCC
gRNAEmerinRv2	AAACGGCGGACTCGGGCGTGGGCGC
HA2FLYWCH1NotI-Fw	CCGCGGCCGCACAGGCCCTGGGTCCCGGGATGCCCCT
HA2FLYWCH1AscI-Rv	CCGGCGCGCCGGAACGGGAAGGCTAGGTTAGCTAA
TurboNheIfrag-F	GCAGGATAGAAAGCTAGCAGGCATCCTGG
TurboNheIfrag-R	CCAGGATGCCTGCTAGCTTTCTATCCTGC
TurboNotI-R	CCGCGGCCGCGTCGGCCCTGCTGAATTCCTTTTC
MiniTurbogoodNheI-F	GACTACGCAGCTAGCATCCCGCTGCTG
gRNAFLYWCH1Fw	CACCGGGGTGCTGAGCGTGGCCTGA
gRNAFLYWCH1Rv	AAACTCAGGCCACGCTCAGCACCCC
FLYWCH1EcorI-Fw	CCGAATTCGGATGCCCCTGCCCGAGCCCAGCGAG
FLYWCH1KpnI-Rv	CCGGTACCTCACTGGGACTCGCCATCCAGTCTGAC
3xV5	GCTCCGCGGCCGCAATGATCCCTAACCCTCTCCTCGGTCTCGATATTCC<br>AAATCCCTTACTCGGCCTAGACATACCGAACCCATTGCTTGGACTGGAT<br>GGCGGGGGAGGTTCTGGTGGGGGCGGATCAGCGGCCGCACAGGC
HA1FLYWCH1	CCCATATGGTATCTCCTGAAGTGGGGGTGGCCTGGGGCCTCCAGGCTA<br>GGATAGGGACTGGGTCTGCCTGAGCCCAGAAAGGGCTTTCTGCCTCCC<br>AGGCCGTGATGTGGTGCCGTCCTCTGGAAAGGGCTCTGCGTACCAGGC<br>CCACATGCCTGAGACAGGTGCCTGGCGTCCTGGTGAAGCACATGGTTT<br>CCAAAAAGGTCCTGACAAGTCCCTGGGCATCTGAGCAGAAACGGGTCT<br>CAGCTTCCCGCCTCTTCAGCCTCAAGTGTATCACAGGTGTCTGTGCCTC<br>ACTATCCTCGTCTATAACTGGGTCAATAAGAACGTTCTGCCGTCATAAT<br>GTAGGGACTGGAGGGGGGACTGGAAACGCGGGTGGAAACCCTTCGT<br>CCGTGCTCAGCATGTGGCTGAGCTCTTAGTCCATTAGTCACTCTGATGG<br>CCCCTTGTGGAGGGTGTTTATTGTGCACATTTTCCAGATGGGGAAAGT<br>GGGGTTGGATGGGGCTGACGTACCTAAGGCTGCCAGGAGAGGAACTG<br>GCAGAGCAGAAGCCTAGTTCCATCAGGCCCCCGGCCAGTCTTGGCCCC<br>AGCGAGCAGTGAGGCAGCCCCATCTGCCCCACCTCCCTCCCAGATCCA<br>CGTCTAGAGTCCCCCAAGGGCTTCCACCACTGACGGGATTTTGCTTCCT<br>TCCTTAGGACGGAACCACTGCACTCCAGGTTCCTTGCTGGGTGCTGAG<br>CGTGGCCTGACCATGGGG
**Oligonucleotides used for pulldown**	
Neg_FLYWCH1-FW	/5Biosg/ACAGATGTCCCGGTCAATATGAAATGAAGTTCCAATGA
Neg_FLYWCH1-Rv	TCATTGGAACTTCATTTCATATTGACCGGGACATCTGT
MotifFLYWCH1_1-FW	/5Biosg/ACAGATGTCCAATCGAATGGAGTTCCAATGA
MotifFLYWCH1_1-Rv	TCATTGGAACTCCATTCGATTGGACATCTGT
MotifFLYWCH1_2-FW	/5Biosg/ACAGATGTCCAATCGAATGGAATCATCAGTTCCAATGA

MotifFLYWCH1_2-Rv	TCATTGGAACTGATGATTCCATTCGATTGGACATCTGT
MotifFLYWCH1_3-FW	/5Biosg/ACAGATGTCCTCATCATCGAAGTTCCAATGA
MotifFLYWCH1_3-Rv	TCATTGGAACTTCGATGATGAGGACATCTGT
