# Supplementary material for: Off-the-shelf proximity biotinylation for interaction proteomics
Source: Nat Commun. 2021 Aug 18;12:5015. doi: 10.1038/s41467-021-25338-4 (PMC8373943; doi:10.1038/s41467-021-25338-4)
Supplement: Supplementary file 3 — Reporting Summary [file 41467_2021_25338_MOESM3_ESM.pdf]

nature research

Corresponding author(s): Dr. Guido van Mierlo
Prof. Michiel Vermeulen

Last updated by author(s): 2021/07/13

Reporting Summary

Nature Research wishes to improve the reproducibility of the work that we publish. This form provides structure for consistency and transparency in reporting. For further information on Nature Research policies, see our Editorial Policies and the Editorial Policy Checklist.

Statistics

For all statistical analyses, confirm that the following items are present in the figure legend, table legend, main text, or Methods section.

n/a	Confirmed	
☐	☒	The exact sample size (*n*) for each experimental group/condition, given as a discrete number and unit of measurement
☒	☐	A statement on whether measurements were taken from distinct samples or whether the same sample was measured repeatedly
☐	☒	The statistical test(s) used AND whether they are one- or two-sided<br>*Only common tests should be described solely by name; describe more complex techniques in the Methods section.*
☒	☐	A description of all covariates tested
☐	☒	A description of any assumptions or corrections, such as tests of normality and adjustment for multiple comparisons
☐	☒	A full description of the statistical parameters including central tendency (e.g. means) or other basic estimates (e.g. regression coefficient) AND variation (e.g. standard deviation) or associated estimates of uncertainty (e.g. confidence intervals)
☐	☒	For null hypothesis testing, the test statistic (e.g. *F*, *t*, *r*) with confidence intervals, effect sizes, degrees of freedom and *P* value noted<br>*Give P values as exact values whenever suitable.*
☒	☐	For Bayesian analysis, information on the choice of priors and Markov chain Monte Carlo settings
☒	☐	For hierarchical and complex designs, identification of the appropriate level for tests and full reporting of outcomes
☐	☒	Estimates of effect sizes (e.g. Cohen's *d*, Pearson's *r*), indicating how they were calculated

Our web collection on statistics for biologists contains articles on many of the points above.

Software and code

Policy information about availability of computer code

Data collection

Microscopy images in the current study were acquired using an LSM900 microscope from Zeiss. Mass spectrometry data was collected on an Orbitrap Exploris, a LTQ-Orbitrap-Fusion or a LTQ-Orbitrap Q-Exactive HFX mass spectrometer from Thermo, which were operated using the software supplied by the manufacturer (Thermo Excalibur version 4.3 or higher and Tune method version 2.9 or higher).

Data analysis

For mass spectrometry, data was analyzed using MaxQuant version 1.5.1.0 and searched against the human proteome downloaded from UniProt. Further data processing was performed using Perseus 1.5.0.15. Protein interaction networks were generated using Cytoscape version 3.8.2.

Biotin ChIP-seq data was aligned using bwa-mem2 version 0.7.17 against the hg38 genome build and duplicates were marked using picard tools (version 1.129). FLYWCH1/IgG ChIP-seq was aligned using the seq2science pipeline (10.5281/zenodo.3921913). Parameters were fastp as trimmer, bwa-mem2 as aligners, minimal map quality of 30. For all files, peaks were called using macs2 version 2.2.7.1 (Zhang, Genome Biology, 2008) with q-value 0.001 for H3K9me3 tracks and q 0.05 for FLYWCH1 and IgG ChIP-seq. Motif analyses were performed using Homer v4.11. Heatmap visualizations were generated using fluff version 3.0.3. Karyogram visualizations were generated using karyoploteR version 1.18.0. Intersections with repeat elements were performed using the bedtools suite version 2.27.1.

Immunofluorescence images were processed using Fiji software version 2.1.0.

For manuscripts utilizing custom algorithms or software that are central to the research but not yet described in published literature, software must be made available to editors and reviewers. We strongly encourage code deposition in a community repository (e.g. GitHub). See the Nature Research guidelines for submitting code & software for further information.

Data

Policy information about availability of data

All manuscripts must include a data availability statement. This statement should provide the following information, where applicable:

- Accession codes, unique identifiers, or web links for publicly available datasets
- A list of figures that have associated raw data
- A description of any restrictions on data availability

The mass spectrometry proteomics data have been deposited to the ProteomeXchange Consortium via the PRIDE (Perez-Riverol, NAR, 2019) partner repository with the dataset identifier PXD025012. ChIP-sequencing data genereated in this study can be found under the reference number GSE169317 in GEO database. Reference H3K9me3 ChIP-seq data was download from the GEO database using accession number GSE86814.

Field-specific reporting

Please select the one below that is the best fit for your research. If you are not sure, read the appropriate sections before making your selection.

☒ Life sciences ☐ Behavioural & social sciences ☐ Ecological, evolutionary & environmental sciences

For a reference copy of the document with all sections, see nature.com/documents/nr-reporting-summary-flat.pdf

Life sciences study design

All studies must disclose on these points even when the disclosure is negative.

Sample size	No statistical methods were used to calculate necessary sample size. All experiments were performed in triplicate, which allows outlier statistics to determine protein enriched in a specific condition relative to a control condition.
Data exclusions	No data was excluded from analyses.
Replication	All mass spectrometry experiments were performed in triplicates and most experiments were repeated independently at least once. Immunofluorescence experiments were performed independently at least twice.
Randomization	Randomization is not required as all samples undergo the same experimental treatment with the exception of the added primary antibody.
Blinding	Investigators were not blinded in this study, as all samples undergo the same experimental treatment with the exception of the added primary antibody.

Reporting for specific materials, systems and methods

We require information from authors about some types of materials, experimental systems and methods used in many studies. Here, indicate whether each material, system or method listed is relevant to your study. If you are not sure if a list item applies to your research, read the appropriate section before selecting a response.

Materials & experimental systems

n/a	Involved in the study	
☐	☒	Antibodies
☐	☒	Eukaryotic cell lines
☒	☐	Palaeontology and archaeology
☒	☐	Animals and other organisms
☒	☐	Human research participants
☒	☐	Clinical data
☒	☐	Dual use research of concern

Methods

n/a	Involved in the study	
☐	☒	ChIP-seq
☒	☐	Flow cytometry
☒	☐	MRI-based neuroimaging

Antibodies

Antibodies used	The antibodies used for this study were IgG rabbit antibody (12-370, Millipore), a-beta-actin (A1978, SIGMA), a-BRG1 (Bethyl, A300-813A), a-H3K9me3 (Abcam, ab8898), a-emerin (10351-1-AP, Proteintech), a-V5 (P/N 46-0705, Invitrogen), a-FLAG (Sigma, F3165), a-INCENP (Active Motif, 39260) and FLYWCH1 (Novus Biologicals, NBP1-85041). For ProtA-Turbo experiments, all antibodies (stock of 1 ug/ul) were diluted 1:100. For ChIP, a-V5 or a-IgG antibodies were diluted 1:150. For western blot, dilutions were a-actin (A1978, SIGMA) diluted 1:5000, a-BRG1 (Bethyl, A300-813A) diluted 1:1000, a-H3K9me3 (Abcam, ab8898) diluted 1:1000, a-emerin (10351-1-AP, Proteintech) diluted 1:1000, a-V5 antibody (P/N 46-0705, Invitrogen) diluted 1:1000, a-FLAG (Sigma, F3165) diluted 1:1000, a-SENP1 (Novus Biologicals, NBP2-55420) diluted 1:1000, a-SENP7 (Bethyl laboratories, A302-995A-T) 1:1000, a-HP1a (Cell Signaling, 2616S) 1:1000, and HRP-conjugated secondary antibody (Dako) diluted 1:1000. Biotin was stained with HRP-Streptavidin (5911, Invitrogen) diluted 1:1000. Secondary antibodies were Polyclonal Rabbit anti-Mouse Immunoglobulins-HRP (Dako, P0260) or

	Polyclonal Swine anti-Rabbit Immunoglobulins-HRP (Dako, P0399), which were used 1:1000. For immunofluorescence, all primary antibodies were used 1:150. Secondary antibodies were anti-Rabbit (Alexa Fluor (Life Technologies A11004), dilution 1:1000) and Avidin-FITC (ThermoFisher Scientific A821) dilution 1:200.
Validation	H3K9me3 antibody(Abcam, ab8898): validated by the vendor using a peptide competition assay.<br>BRG1 antibody (Bethyl,A300-813A): validated by the vendor (size validation on western blot and targeted / control immunoprecipitations followed by western blot). Also validated in: Xue et al., 2019, Nature Communications.<br>Emerin antibody (Proteintech, 10351-1-AP): validated by the vendor (correct localization of immunofluorescent signal in nuclear membrane, correct western blot size, Emerin or IgG immunoprecipitation followed by western blot for the Emerin antibody). Also validated in: Elizabeth R. Smith et al., BMC Cell Biology, 2017.<br>Actin antibody (SIGMA, A1978): validated by the vendor (localization in cells using immunofluorescence, correct size on western blot)<br>V5 antibody (Invitrogen, P/N 46-0705): validated by the vendor (immunofluorescence and western blot of wild-type or V5-tagged cell lines).<br>FLAG (Sigma, F3165): western blot validated by the vendor.<br>SENP1 (Novus Biologicals, NBP2-55420): validated by the vendor (immunofluorescence and western blot).<br>SENP7 (Bethyl laboratories, A302-995A-T): validated by the vendor (correct western blot size, SENP7 or IgG immunoprecipitation followed by western blot for the Emerin antibody). Also validated in: González-Prieto et al., Cell cycle, 2015.<br>HP1a (Cell Signaling, 2616S): validated by the vendor (western blot correct size, immunofluorescence staining showing punctuate foci in the nucleus, ChIP-qPCR for two repetitive, H3K9me3-marked genomic loci in ESCs).

Eukaryotic cell lines

Policy information about cell lines

Cell line source(s)	HeLa, U937 and MCF7 cells; ATCC<br>Primary human fibroblasts; Coriell Institute (# AG08469)<br>ESCs: Thomas Jenuwein lab
Authentication	None of the cell lines where authenticated.
Mycoplasma contamination	Cell lines were tested negative for Mycoplasma using a MycoAlert TM Mycoplasma Detection kit from Lonza (LT07-118).
Commonly misidentified lines<br>(See ICLAC register)	No commonly misidentified cell lines were used.

ChIP-seq

Data deposition

☒ Confirm that both raw and final processed data have been deposited in a public database such as GEO.

☒ Confirm that you have deposited or provided access to graph files (e.g. BED files) for the called peaks.

Data access links<br>*May remain private before publication.*	Go to https://www.ncbi.nlm.nih.gov/geo/query/acc.cgi?acc=GSE169317
Files in database submission	pA-Turbo targeted H3K9me3 biotin ChIP rep 1<br>pA-Turbo targeted H3K9me3 biotin ChIP rep 2<br>pA-Turbo targeted IgG biotin ChIP rep 1<br>pA-Turbo targeted IgG biotin ChIP rep 2<br>Flywch1 ChIP-seq rep1<br>Flywch1 ChIP-seq rep2<br>IgG ChIP-seq rep1<br>IgG ChIP-seq rep2
Genome browser session<br>(e.g. UCSC)	Not applicable. The data submitted to GEO includes bigwig files that can be directly uploaded to the genome browser (hg38) for visual inspection of the data.

Methodology

Replicates	Every experiment was performed in duplicate. The replicates for the biotin chips were independent biological samples, while the FLYWCH1/IgG ChIP-seq was performed in duplicate on the same chromatin batch.
Sequencing depth	#File name #reads #mapped reads # read length #type<br>pA-Turbo targeted H3K9me3 biotin ChIP rep 1 49006168 46525217 42 paired-end<br>pA-Turbo targeted H3K9me3 biotin ChIP rep 2 50052316 47466809 42 paired-end<br>pA-Turbo targeted IgG biotin ChIP rep 1 48502872 36661848 42 paired-end<br>pA-Turbo targeted IgG biotin ChIP rep 2 42039666 30105417 42 paired-end<br>Flywch1 ChIP-seq rep1 64564710 45447520 38 paired-end<br>Flywch1 ChIP-seq rep2 59464658 35361377 38 paired-end<br>IgG ChIP-seq rep1 70617130 57586349 38 paired-end

	IgG ChIP-seq rep2 76936554 62855028 38 paired-end
Antibodies	pA-Turbo targeted H3K9me3 biotin ChIP rep 1 Targeting Ab: a-H3K9me3 (Abcam, #Cat:ab8898). ChIP with Dynabeads M280 Strepatividin 11205D<br>pA-Turbo targeted H3K9me3 biotin ChIP rep 2 Targeting Ab: a-H3K9me3 (Abcam, #Cat:ab8898). ChIP with Dynabeads M280 Strepatividin 11205D<br>pA-Turbo targeted IgG biotin ChIP rep 1 Targeting Ab: Rabbit IgG (Millipore, #Cat: 12-370). ChIP with Dynabeads M280 Strepatividin 11205D<br>pA-Turbo targeted IgG biotin ChIP rep 2 Targeting Ab: Rabbit IgG (Millipore, #Cat: 12-370). ChIP with Dynabeads M280 Strepatividin 11205D<br>Flywch1 ChIP-seq rep1 a-V5 (Invitrogen, #Cat: P/N46-0705)<br>Flywch1 ChIP-seq rep2 a-V5 (Invitrogen, #Cat: P/N46-0705)<br>IgG ChIP-seq rep1 Rabbit IgG (Millipore, #Cat: 12-370)<br>IgG ChIP-seq rep2 Rabbit IgG (Millipore, #Cat: 12-370)
Peak calling parameters	Macs2 was used for peak calling in all instances, with a q-value of 0.05.
Data quality	IgG targeting was used as control. For biotin chip-seqs, no peak calling was performed but mainly involved global analyses (i.e. enrichment of signal on known H3K9me3 domains). For FLYWCH1 and IgG ChIP-seq: IgG#1 - 105 peaks; IgG#2 - 108 peaks; FLYWCH1#1 - 585 peaks; FLYWCH1#2 - 812 peaks. Only peaks that were overlapping in both FLYWCH1 replicates and not present in IgG samples were used for downstream analyses (451 peaks in total).
Software	ChIP-seq mapping was performing using bwa-mem2 (Li, 2013; https://arxiv.org/abs/1303.3997). Reference H3K9me3 ChIP-seq in wildtype HeLa cells was downloaded from GEO (accession number GSE86814 (Timms, Cell Reports, 2016)) and processed in parallel. Duplicate reads were identified and filtered using Picard tools version 1.129 (http://broadinstitute.github.io/picard/). FLYWCH1 and IgG ChIP-seq was processed using the seq2science pipeline (10.5281/zenodo.3921913). For all files, peaks were called using macs2 (Zhang, Genome Biology, 2008). ChIP-seq heatmaps were generated using fluff heatmap (Georgiou, 2016). Motif analysis was performed using Homer (Heinz, Mol. Cell., 2010) using default parameters, genome hg38 and width 200. Kayotype plots were generated in R using karyoploteR (Gel, Bioinformatics, 2017). ChIP-seq tracks were visualized in Integrative Genomics Viewer.
